# No Evidence for Human Monocyte-Derived Macrophage Infection and Antibody-Mediated Enhancement of SARS-CoV-2 Infection

**DOI:** 10.3389/fcimb.2021.644574

**Published:** 2021-04-12

**Authors:** Obdulio García-Nicolás, Philip V’kovski, Ferdinand Zettl, Gert Zimmer, Volker Thiel, Artur Summerfield

**Affiliations:** ^1^ Institute of Virology and Immunology (IVI), Bern, Switzerland; ^2^ Department of Infectious Diseases and Pathobiology, Vetsuisse Faculty, University of Bern, Bern, Switzerland

**Keywords:** human coronaviruses, SARS-CoV-2, COVID-19 convalescent sera, ADE, human monocyte-derived macrophages

## Abstract

Vaccines are essential to control the spread of severe acute respiratory syndrome coronavirus-2 (SARS-CoV-2) and to protect the vulnerable population. However, one safety concern of vaccination is the possible development of antibody-dependent enhancement (ADE) of SARS-CoV-2 infection. The potential infection of Fc receptor bearing cells such as macrophages, would support continued virus replication and inflammatory responses, and thereby potentially worsen the clinical outcome of COVID-19. Here we demonstrate that SARS-CoV-2 and SARS-CoV neither infect human monocyte-derived macrophages (hMDM) nor induce inflammatory cytokines in these cells, in sharp contrast to Middle East respiratory syndrome (MERS) coronavirus and the common cold human coronavirus 229E. Furthermore, serum from convalescent COVID-19 patients neither induced enhancement of SARS-CoV-2 infection nor innate immune response in hMDM. Although, hMDM expressed angiotensin-converting enzyme 2, no or very low levels of transmembrane protease serine 2 were found. These results support the view that ADE may not be involved in the immunopathological processes associated with COVID-19, however, more studies are necessary to understand the potential contribution of antibodies-virus complexes with other cells expressing FcR receptors.

## Introduction

Since the emergence of the severe acute respiratory syndrome coronavirus-2 (SARS-CoV-2) in December 2019 in the Chinese city of Wuhan, the virus has spread globally causing the coronavirus disease 2019 (COVID-19) pandemic. The high morbidity and severity of COVID-19 in some of the affected patients have jeopardized the public health system of affected countries. In addition, the public health measures that have been implemented to control the pandemic have affected the life and economy of millions of people around the world.

In the current situation, the lack of neutralizing antibodies against SARS-CoV-2, allows the virus to spread rapidly in the human population. A global vaccination campaign may have the potential to finally control the pandemic and vaccination programs have started recently. However, a concern is the possibility that vaccination could promote antibody-dependent enhancement (ADE) of SARS-CoV-2 infection which could be associated with enhancement of the disease (Lee et al., 2020).

The underlying mechanism of ADE of infection is based on the interaction between virion-antibody complexes and Fc gamma receptors (FcγR) that are expressed by cells of the immune system such as macrophages. The binding of virion-antibody complexes to Fc receptors could result in their uptake into the cells by receptor-mediated endocytosis leading to potential infection of the cells (Taylor et al., 2015). Despite speculation and alarming about this possibility at the time of initiation of our work, no data were published specifically addressing ADE of SARS-CoV-2 (Lee et al., 2020; Rogers et al., 2020). With this in mind, the present study aimed to investigate whether immune sera from convalescent COVID-19 patients would enhance SARS-CoV-2 infection and promote secretion of pro-inflammatory cytokines production by human macrophages. To this end we performed a comparative study on the susceptibility of human macrophages to infection with human coronavirus 229E (HCoV-229E), Middle East respiratory syndrome coronavirus (MERS-CoV), SARS-CoV and SARS-CoV-2 and the inflammatory cytokine response of these cells. Potential ADE of infections by SARS-CoV and SARS-CoV-2 were studied using immune sera from convalescent COVID-19 patients.

## Material and Methods

### Ethics Statement

Buffy coats from anonymous healthy blood donors were obtained from the regional transfusion blood service of the Swiss Red Cross (SRC) (Bern, Switzerland). The use of buffy coats was approved by the SRC review board. All serum samples employed in this study were collected following the guidance of the Act on Medical Devices (MPG guideline 98/79/EC) for the collection of human residual material to evaluate suitability of an *in vitro* diagnostic medical device (§24). For this study an informed consent and ethical approval was not needed because only leftovers of serum samples for diagnostic laboratory procedures were used.

### Cells

Vero cells (E6 and B4 lineages, African Green monkey kidney epithelial cells) and Huh-7 cells (human hepatocellular carcinoma cells) were cultured in Dulbecco’s minimal essential medium (DMEM; Life Technologies), supplemented with 10% fetal bovine serum (FBS), non-essential amino acids (Life Technologies), penicillin-streptomycin (Gibco) and HEPES (Gibco). A549 (adenocarcinomic human alveolar basal epithelial cells) stably transfected with angiotensin-converting enzyme 2 (ACE2) and transmembrane protease serine 2 (TMPRSS2) were purchased from Invivogen (Toulouse, France) and cultured in minimal essential medium (MEM) supplemented with 10% FBS, 0.5 μg/ml of puromycin (Invivogen) and 300 μg/ml of hygromycin B (Invivogen). *A. albopictus* C6/36 cells (ATCC^®^ CRL-1660™) were cultured in MEM (Gibco) supplemented with 100 mM of sodium pyruvate (Gibco), non-essential amino acids and 10% FBS

For the production of human monocyte derived macrophages (hMDM), peripheral blood mononuclear cells (PBMCs) were isolated from buffy coats by density gradient centrifugation on Ficoll-Paque™ PLUS (1.077 g/L; GE healthcare). Then, monocytes were sorted using anti-CD14 beads as proposed by the manufacturer (Miltenyi Biotech GmbH), and seeded in 24 well plates at 2.5 x 10^5^ cells/well in 500 µl of Roswell Park Memorial Institute (RPMI) 1640 medium (Gibco) and kept at 37°C and 5% CO_2_ atmosphere for one hour. Non-adherent cells were removed and 500 µl of RPMI 1640 supplemented with 10% of FBS (Gibco), GlutaMAX (Gibco), penicillin-streptomycin (Gibco) and human M-CSF (100 ng/ml; Miltenyi Biotec) were added. The cells were cultured for six days at 37 °C and 5% CO_2_. The full medium complemented with M-CSF was replaced every 48 to 72 hours.

### Viruses

A collection of different coronaviruses was employed for the experiments of the present study including the human coronavirus 229E [HCoV-299E; (Thiel et al., 2001)], Middle East respiratory syndrome coronavirus [MERS-CoV, strain EMC/2012; (van Boheemen et al., 2012)], SARS-CoV [Frankfurt-1; (Thiel et al., 2003)] and SARS-CoV-2 (SARS-CoV-2/München-1.1/2020/929) kindly provided by Daniela Niemeyer, Marcel Müller, and Christian Drosten (Charité, Berlin, Germany). HCoV-299E was propagated in Huh-7 cells in DMEM supplemented with 5% of FBS and non-essential amino acids at 33°C. MERS-CoV was propagated in Vero B4 cells in MEM supplemented with 2% of FBS and non-essential amino acids at 37°C. For the propagation of SARS-CoV and SARS-CoV-2 Vero E6 cells in MEM supplemented with 2% of FBS and non-essential amino acids at 37°C was employed. All coronavirus titrations were performed by end point dilution (ten-fold serial dilutions of viral supernatants) taking advantage of the virus-induced cytopathic effect that was apparent 56 to 72 hours post infection (hpi). Virus titers were expressed as 50% tissue culture infective dose per ml (TCID_50_/ml). As a positive control for ADE of infection, we used Japanese encephalitis virus (JEV) (Laos strain; GenBank CNS769_Laos_2009; kindly provided by Prof. Remi Charrel, Aix-Marseille Université, Marseille, France) with immune sera previously described (Garcia-Nicolas et al., 2017).

### Infection With Coronaviruses

Vero E6 cells or hMDM were incubated for 1.5 h at 39°C or 37°C with the respective virus using a multiplicity of infection (MOI) of 1 TCID_50_ per cell, including mock control. Subsequently, the virus inoculum was removed, the cells washed three times with warm phosphate buffered saline (PBS), and RPMI medium supplemented with 2% FBS was added to the cells. As a positive control for the induction of pro-inflammatory cytokines either 1 µg/ml of lipopolysaccharide (LPS; Sigma-Aldrich) or 10 μg/ml of polyinosinic-polycytidytic acid (poly I:C, Sigma-Aldrich) were added to the cell culture medium. As indicated for each experiment, after 24, 48 or 72 h, supernatants were collected and stored at -70°C.

### ACE2/TMPRSS2 Determination

Human macrophages and A549 cells stably transfected with human ACE2 and human TMPRSS2 were harvested using TrypLETMSelect (Gibco) for 20 min at room temperature and washed with CellWash solution (Becton Dickinson). Subsequently, the cells were stained for 20 min with anti-human TMPRSS2 monoclonal antibody (P5H9-A3, Santa Cruz biotechnology) and anti-human ACE2 antibody (A20069, BioLegend) in CellWash. After a washing the cells with CellWash, they were incubated for 10 min with anti-rat Alexa 488 fluorochrome conjugate (ThermoFisher Scientific) and Alexa Fluor 647 conjugated anti-mouse IgG1 (ThermoFisher Scientific). Finally, cells were analyzed by flow cytometry using a FACSCantoII (Becton Dickinson). Data analysis was performed with Flowjo V.9.1 software (Treestars, Inc.). Dead cells were excluded by electronic gating in forward/side scatter plots, followed by exclusion of doublets.

### Antibody-Dependent Enhancement of Infection

A collection of sera from COVID-19 convalescent patients from a previously published work was employed for the present study (Zettl et al., 2020). This included sera with a broad range of neutralization titers against SARS-CoV-2 (ND_50_ <1:10; 1:20; 1:160; 1:240 and 1:2560). In order to test the ADE potential of these sera, different serum dilutions (1:10; 1:100; 1:1000 and 1:10000) were incubated for 30 min at 37°C with an equal volume of viral suspension (SARS-CoV or SARS-CoV-2) corresponding to a MOI of 1 TCID_50_/cell. Thereafter, the virus/serum mixtures were added to human macrophages or Vero E6 cells and incubated for 30 min at 37°C and 5% CO_2_ atmosphere. The cells were washed three times with PBS before fresh medium was added. As a control for ADE of infection, we employed a serum from immunized pigs known to have a high capacity of inducing ADE of infection for JEV in macrophages (Garcia-Nicolas et al., 2017). The serum (ND_50_ of 1:160 for JEV Laos) was serially diluted (1:10; 1:100; 1:1000 and 1:10000) and incubated at 37°C for 30 min with JEV Laos at a MOI of 1 TCID_50_/cell. Porcine naïve serum was included as control. After that, virus/serum mixtures were added on hMDM and incubated for 30 min at 37°C, washed off and fresh medium was added. After 24 h of incubation at 37°C viral infectivity was determined by means of flow cytometry as described below.

### Determination of Infected Cells

For immunofluorescence microscopy, cells were fixed with 4% formaldehyde for 10 min at room temperature, washed with PBS, and permeabilized with 0.3% saponin (PanReac AppliChem). The permeabilization procedure was performed for 20 min on ice in the presence of J2 monoclonal antibody directed to dsRNA (English and Scientific Consulting), or some experiments a rabbit antibody directed to the SARS-CoV nucleocapsid (N) protein (Rockland-inc) was included in this step. Subsequently, the cells were washed with 0.1% saponin, and cells were incubated for 20 min on ice with Alexa Fluor 488 conjugated anti-mouse IgG2a (ThermoFisher Scientific) or with Alexa Fluor 546 conjugated anti-rabbit (ThermoFisher Scientific) in 0.3% saponin. Cells were washed once with PBS prior to incubation with 4′,6-diamidino-2-phenylindole (DAPI; Sigma) for 5 min at 37 °C. Finally, the percentage of infected cells was determined by enumerating the dsRNA positive cells in 10 fields/well using an Axio Observer Z1 inverted microscope equipped with a Zeiss Colibri Illuminator (CarlZeiss) and digital imaging Zeiss software (AxioVision, v4). All generated images were analyzed using ImageJ software.

For the determination of infected cells by flow cytometry, macrophages and Vero E6 cells were harvested using TrypLETMSelect (Gibco) for 20 min at room temperature and fixed with 4% (w/v) formaldehyde. Thereafter, the cells were permeabilized/stained for 20 min on ice with 0.3% (*w*/*v*) saponin in PBS in the presence of a rabbit antibody directed to the SARS-CoV N protein (Rockland-inc). For some experiments we used an anti-dsRNA monoclonal antibody (J2, English and Scientific Consulting). For the determination of JEV infected cells anti-flavivirus E protein mAb 4G2 (IgG2a) was employed as primary antibody. The cells were subsequently washed, incubated for 10 min with anti-rabbit Alexa 488 or Alexa Fluor 647-conjugated anti-mouse IgG2a (ThermoFisher Scientific) and analyzed by flow cytometry (FACSCantoII, Becton Dickinson). For analysis, Flowjo V.9.1 software (Treestars, Inc.) was used. Dead cells were excluded by electronic gating in forward/side scatter plots, followed by exclusion of doublets.

### Determination of Cytokines

Cell culture supernatants were analyzed for the presence of the following cytokines: Tumor necrosis factor (TNF), interferon beta (IFN-β), interleukin 6 (IL-6) and IL-1β were quantified by ELISA (R&D Systems) following the manufacturer’s instructions. Detection limits were 30 pg/ml for TNF, 10 pg/ml for IFN-β, 4 pg/ml for IL-1β and 9 pg/ml for IL-6.

### Statistics

**Figure 1:**
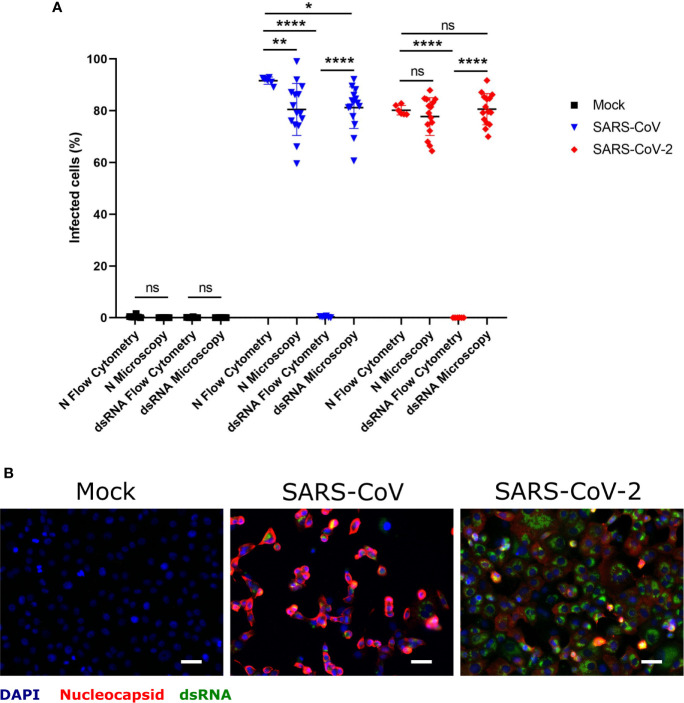
Determination of SARS-CoV and SARS-CoV-2 infected hMDM by immunolabeling for dsRNA and N protein. Vere E6 cells were infected with SARS-CoV and SARS-CoV-2 at MOI 1 TCID_50_/cell, and after 24 hpi dsRNA and N protein were labeled with specific antibodies. The nuclei were stained with DAPI. Then positive cells for dsRNA and N were quantified either by flow cytometry or immunofluorescence microscopy **(A)**. In **(B)** example of representative images acquired by fluorescence microscopy is shown. The scale bar represent 40 µm. The data are from three independent experiments. Statistically significant differences between the conditions are indicated by asterisks (ns indicates non-statistical differences, *p < 0.05, **p ≤ 0.002 and ****p ≤ 0.0001).

For the generation of figures and data analyses the GraphPad Prism 8 Software (GraphPad Software, Inc.) was employed. All experiments were independently performed 3 to 6 times with cells from different donors, and each experiment was run in triplicates. For viral titrations, differences between groups were assessed using a Kruskal–Wallis test, and for individual differences the Mann–Whitney *U*-test with Bonferroni correction as *post hoc* was employed. For group differences in the percentages of infected cells and levels of cytokines expression comparisons, a one-way ANOVA test with Bonferroni correction as *post hoc* was performed. Correlation analysis between infected cells, viral titers, and expressed cytokines were calculated by Spearman’s Rho analysis; a correlation between two different factors was considered relevant with R^2^ >0.5. A *p* value lower than 0.05 was considered statistically significant for every analyzed data. In figure 1, different superscript letters indicate a significant difference (p < 0.05) between the conditions. For the table 2 ^*^ indicates *p* <0.05, ^**^ *p* ≤0.002, ^***^ *p* ≤0.001 and ^****^ *p* ≤0.0001.

## Results

### Visualization of Coronavirus Replication by Immunolabeling of dsRNA

Taking into account that dsRNA is a replication intermediate located in double-membrane vesicles during coronavirus replication (Wolff et al., 2020), coronavirus-infected cells may be specifically detected by antibodies to dsRNA rather than by antibodies directed to viral proteins. In order to evaluate the suitability of dsRNA immunolabeling for this purpose, Vero E6 cells infected with either SARS-CoV or SARS-CoV-2 at MOI of 1 TCID_50_/ml, were double-stained for dsRNA and N protein, and analyzed by immunofluorescence microscopy or flow cytometry (**Figure 1**). While dsRNA staining allowed us to identify infected cells by immunofluorescence microscopy to a similar degree as N protein labeling (**Figures 1A, B**), flow cytometry only worked when the N protein was labeled (**Figure 1A**). This experiment demonstrated that immunolabeling of dsRNA allows the identification of cells infected with different coronaviruses by immunofluorescence microscopy in the absence of antibodies specifically recognizing viral proteins. On the other hand, the labeling of N protein in combination with flow cytometry is an efficient way of detecting cells infected by SARS-CoV or SARS-CoV-2.

### Human Coronaviruses Differ in Their Ability to Infect hMDM

**Figure 2:**
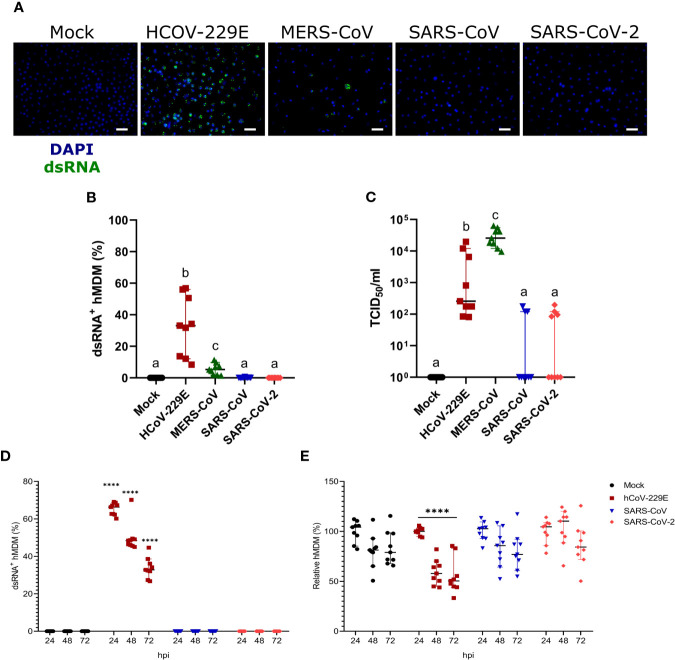
Susceptibility of hMDM to different human coronaviruses. Human MDM were inoculated with different coronaviruses (hCoV-229E, MERS-CoV, SARS-CoV-1 and SARS-CoV-2) using an MOI of 1 TCID_50_/cell. Mock-infected cells were included as controls. After incubating the cells for 1.5 hours, the inoculum was removed, the cells washed, and fresh medium added. At 24 hpi, dsRNA in the cells was detected with a specific antibody and nuclei were stained with DAPI; the scale bar represents 40 µm. **(A)** The percentage of dsRNA-positive hMDM was calculated for 10 fields per condition **(B)**. In **(C)** virus titers are shown. The same experiment was repeated with hCoV-229E, SARS-CoV-1 and SARS-CoV-2, and infected cells were quantified at 24, 48 and 72 hpi **(D)**. The relative number of total hMDM per well was calculated taking as reference the number of cells at 24 hpi **(E)**. The data from three independent experiments run in triplicates are shown in each panel. Statistically significant differences between the conditions are indicated by different superscript letters in **(B, C)** (p < 0.05), and by asterisks in **(D, E)** (****p ≤ 0.0001).

Infection of hMDM with HCoV-299E, MERS-CoV, SARS-CoV and SARS-CoV-2 at MOI of 1 TCID_50_/cell demonstrated high susceptibility to infection with the common cold virus HCoV-229E, low susceptibility to infection with the highly pathogenic coronavirus MERS-CoV, and resistance to infection by SARS-CoV and SARS-CoV-2, in terms of dsRNA immunostaining(**Figures 2A, B**). Quantification of the number of infectious virus particles in the cell culture supernatants showed that only HCoV-229E and MERS-CoV replicated efficiently in hMDM (**Figure 2C**). Although HCoV-229E showed higher percentages of infected hMDM (32.79% ± 18.79 SD) the highest virus titers were found in the cell culture supernatant of macrophages infected with MERS-CoV (**Figure 2C**). Nevertheless, it has to be taken into consideration that all experiments were performed at 37°C although the optimal temperature for HCoV-229E is 33°C (Dijkman et al., 2013). Viral titers of SARS-CoV and SARS-CoV-2 were not statistically significantly different compared to the mock control. The background signal detected for some wells might be due to remaining viral particles from the inoculum that stayed bound to the hMDM surface and were not removed by washing the cells.

We also tested infectivity of hMDM to SARS-CoV or SARS-CoV-2 at later time points, including 48 and 72 hpi. While HCoV-229E efficiently infected hMDM neither SARS-CoV nor SARS-CoV-2 were able to infect hMDM at any of the time points (**Figure 2D**). Moreover, only HCoV-299E but not SARS-CoV and SARS-CoV-2-infected hMDM showed a significant decrease of the number of cells, indicating a virus-induced cytopathogenic effect (**Figure 2E**).

### Human MDM Produce Cytokines Following Infection With HCoV-229E

**Figure 3:**
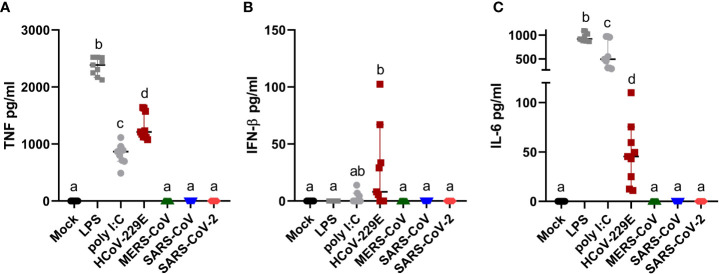
Human MDM immune response after coronavirus infection. Human MDM were inoculated with different coronaviruses (hCoV-229E, MERS-CoV, SARS-CoV-1 and SARS-CoV-2) as described before. Mock-infected cells or cells treated with LPS or poly I:C served as controls. After 24 hpi TNF **(A)** IFN-β **(B)** and IL-6 **(C)** were determined in the cell culture supernatants. The data from three independent experiments run in triplicates are shown. Different superscript letters indicate a significant difference (p < 0.05) between the conditions.

Human MDM infected by HCoV-229E, but not by MERS-CoV, SARS-CoV or SARS-CoV-2, secreted TNF, and low levels of both IFN-β and IL-6 (**Figure 3**). None of the tested coronaviruses induced secretion of IL-1β. Taking into consideration that viral RNA might induce innate immune responses, we tested the correlation between the percentage of dsRNA positive cells, viral titers and level of secreted cytokines. The results found a clear association between the percentage of infected cells and secreted cytokine levels but not with viral titers (**Table 1**).

**Table 1:** Correlation analysis between dsRNA positive hMDM, infectious virus titers and pro-inflammatory cytokines.

R^2^	Viral titer	TNF	IFN-β	IL-6
**dsRNA**	0.015	0.821^****^	0.682^****^	0.892^****^
**Viral titer**	–	0.004	0.006	0.001
**TNF**		–	0.490^****^	0.795^****^
**IFN-β**			–	0.729^****^

^****^p ≤ 0.0001.

### Human MDM Express SARS-CoV-2 Receptor ACE2 but No or Low Levels of TMPRSS2

**Figure 4:**
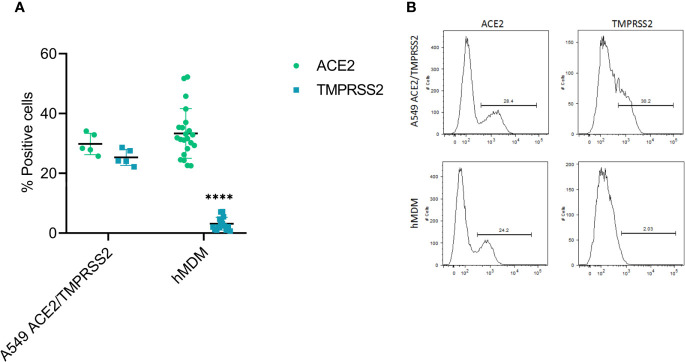
ACE2 and TMPRSS2 expression in hMDM. After 6 days of differentiation ACE2 and TMPRSS2 were immunolabeled with specific antibodies and positive cells were assessed by flow cytometry. A549 cells transfected with ACE2 and TMPRSS2 were used as control **(A)**. Representative histograms for each marker in the analyzed cells are shown **(B)**. The data from 5 different human donor hMDM run in triplicates are shown. Statistically significant differences in the expression of each marker between both cell types are marked by asterisks (****p ≤ 0.0001).

First, as hMDM were resistant to infection by SARS-CoV-2, we assessed the expression levels of the viral cell receptor ACE2, as well as TMPRSS2, a serine protease involved in the proteolytic activation of the spike protein (Hoffmann et al., 2020; Shang et al., 2020). For that, double immunolabeling of cell ACE2 and TMPRSS2 was performed in hMDM after differentiation, and their expression was assessed by flow cytometry (**Figure 4**); A549 cells transfected with ACE2 and TMPRSS2 were used as positive control. This experiment showed that hMDM express high levels of ACE2 (33.3% ± 8.25SD; **Figure 4A**) comparable to the A549 cells transfected with ACE2 and TMPRSS2 (29.8% ± 3.55SD; **Figure 4A**). On the other hand, the percentage of TMPRSS2 positive hMDM was very low (3.03% ± 2.17SD; **Figure 4A**) when compared to the A549 cells transfected with ACE2 and TMPRSS2 (25.3% ± 2.66SD; **Figure 4A**). These results indicate that although hMDM express the SARS-CoV-2 receptor ACE2, the lack of expression of TMPRSS2 might prevent infection.

### Antibodies From Convalescent COVID-19 Patients Neither Induce Antibody-Dependent Enhancement of Infection of hMDM With SARS-COV-2 Nor Promote Cytokine Responses

First, as a positive control for ADE in hMDM, sera from immunized pig that were previously demonstrated to induce a high ADE of JEV in porcine MDM (Garcia-Nicolas et al., 2017) were used. Despite the low binding of porcine IgGs to human FcR (Antonsson and Johansson, 2001), this experiment showed that such JEV immune complexes can enhance the infection of hMDM (**Supplementary Figure 1**) compared with both the JEV Laos control for the infection and the same dilution for the naïve porcine serum. This experiment showed the validity of the methodology to test ADE of infection in hMDM.

Next, selected COVID-19 sera with a neutralization titer below 1:10 and with a neutralization titer of 1:240, were diluted serially from 1:10 to 1:1000, mixed with SARS-CoV and SARS-CoV-2, and then added to Vero E6 cells. Both sera demonstrated a dilution-dependent inhibition of SARS-CoV-2 infection. In addition, a cross-reactivity of COVID-19 convalescent patient sera with SARS-CoV-1 was observed, confirming previous reports (Zettl et al., 2020) (**Table 2**).

**Table 2:** Summary data for virus neutralization on Vero E6 cells and antibody-dependent enhancement of infection or cytokine response by hMDM for SARS-CoV and SARS-CoV-2-antibody complexes”+” detected; “-” not detected; “NT” not tested.

			Neutralization (% N^+^ Vero E6 cells n = 3)		ADE (% N^+^ hMDM n = 6)		Cytokine response (hMDM n = 6)	
			SARS-CoV	SARS-CoV-2	SARS-CoV	SARS-CoV-2	TNF (pg/ml)	IFN-β (pg/ml)
		Infection CTRL (%)	34 (± 1.5)	22.17 (± 2.11)	–	–	–	–
**Immune serum**	**Neutralizing titer**	Serum dilution						
**A**	< 1:10	1:10	10.41 (± 0.86)****	7.07 (± 0.23)****	–	–	–	–
		1:100	29.67 (± 3.99)	15.26 (± 1.92)****	–	–	–	–
		1:1000	36.57 (± 2.59)	23.40 (± 2.55)	–	–	–	–
		1:10000	34.23 (± 2.41)	32.93 (± 0.25)	–	–	–	–
**B**	1:20	1:10	NT	NT	NT	–	–	–
		1:100	NT	NT	NT	–	–	–
		1:1000	NT	NT	NT	–	–	–
		1:10000	NT	NT	NT	–	–	–
**C**	1:160	1:10	NT	NT	NT	–	–	–
		1:100	NT	NT	NT	–	–	–
		1:1000	NT	NT	NT	–	–	–
		1:10000	NT	NT	NT	–	–	–
**D**	1:240	1:10	10.08 (± 1.06)****	0.25 (± 0.26)****	–	–	–	–
		1:100	25.63 (± 1.63)***	2.25 (± 0.34)****	–	–	–	–
		1:1000	31.6 (± 0.72)	12.07 (± 0.58)****	–	–	–	–
		1:10000	32.93 (± 0.25)	18.73 (± 1.19)*	–	–	–	–
**E**	1:2560	1:10	NT	NT	NT	–	–	–
		1:100	NT	NT	NT	–	–	–
		1:1000	NT	NT	NT	–	–	–
		1:10000	NT	NT	NT	–	–	–

Statistically significant differences compared to the control are indicated as: * for *p* < 0.05, *** for *p* ≤ 0.001 and **** for p ≤ 0.0001.

Using the same approach, a larger collection of sera from COVID-19 convalescent patients with neutralization titers ranging from <10 to 1:2560 was incubated at different concentrations with SARS-CoV and SARS-CoV-2 and added to hMDM. Importantly, with none of the tested serum dilutions which went up to 1:10000 infection of hMDM was observed. Moreover, hMDM exposed to virus-antibodies complexes did not secrete any detectable pro-inflammatory cytokines (**Table 2**). These results indicate that the potential uptake of SARS-CoV and SARS-CoV-2 *via* FcR does not result in infection and activation of human macrophages.

## Discussion

A first observation of the present study was that in contrast to the common cold virus hCoV-229E and MERS-CoV, SARS-CoV and SARS-CoV-2 were unable to infect hMDM. The receptor for SARS-CoV-2 is angiotensin-converting enzyme 2 (ACE2) typically expressed on ciliated epithelial cells, goblet cells, type II alveolar pneumocytes as well as other cells from different organs as enterocytes (Sungnak et al., 2020). However, there are conflicting reports on the infection of human macrophages by SARS-CoV. While one study described very limited ACE2 expression by macrophages (Sungnak et al., 2020), another report postulated that the receptor is expressed on tissue resident macrophages (Song et al., 2020). Although we showed in the present study that about 30% of the hMDM express ACE2 under the described culture/differentiation conditions, hMDM were not permissive to SARS-CoV or SARS-CoV-2. Of note, the expression of a truncated ACE2 isoform (dAEC2) has been demonstrated upon IFN stimulation or viral infection (Onabajo et al., 2020). While remaining biologically active, dAEC2 does not facilitate SARS-CoV-2 spike binding and does not serve as an entry receptor. Whether hMDM express dACE2 remains to be formally determined. Following attachment of virions to the cell surface, the spike protein may be proteolytically activated and is able to trigger membrane fusion and release of the viral genome into the cytosol of the host cell. The proteolytic cleavage by TMPRSS2 or related enzymes normally takes place at the cell surface as well as the subsequent membrane fusion (Hoffmann et al., 2020). In the absence of TMPRSS2, SARS-CoV and SARS-CoV-2 may be proteolytically activated following receptor-mediated endocytosis by cathepsin B/L (Hoffmann et al., 2020; Shang et al., 2020). The present study shows that TMPRSS2 is expressed at very low levels in hMDM, which is in line with a previous publication describing very limited expression of TMPRSS2 expression in human macrophages (Sungnak et al., 2020). Therefore, we speculate that this might be an important limiting factor for the viral entry in macrophages. Moreover, recently it has been described that the p41 invariant chain of CD74 (major histocompatibility complex class II) can inhibit the cathepsin-mediated cleavage of viral envelope proteins (Bruchez et al., 2020). As macrophages constitutively express CD74 (Cho and Roche, 2013) the presence of the p41 invariant chain might block the activity of cathepsin B/L contributing to the resistance of hMDM to the infection by SARS-CoV and SARS-CoV-2. In COVID-19 patients, the viral nucleoprotein N has been detected in macrophages from lymphoid organs of COVID-19 patients (Park, 2020), but it is not clear whether this was caused by direct infection or as a consequence of phagocytosis of infected cells.

The infection of hMDM by HCoV-229E is in line with the expression by these cells of aminopeptidase N (CD13), the cellular receptor for HCoV-229E (Yeager et al., 1992). It is also in agreement with previous reports describing the infection of alveolar macrophages by HCoV-229E (Funk et al., 2012). The cellular receptor for MERS-CoV dipeptidyl peptidase-IV (DPP4 also known as CD26) is expressed at low levels by human monocytes and macrophages of healthy donors (Wang et al., 2013; Zhong et al., 2013; Rao et al., 2018; Rao et al., 2019), which could explain MERS-CoV infection of hMDM in our experiments.

Our results are also in line with a previous report demonstrating that following infection with HCoV-229E human macrophages strongly secrete TNF, but also produce IL-6 and some IFN-β (Funk et al., 2012). Finally, we also noticed a poor innate immune response of macrophages following infection with MERS-CoV, confirming a previous report that showed similar results (Zhou et al., 2014).

While writing the present manuscript contradictory data were published claiming that SARS-CoV-2 induces an immune activation of hMDM (Zheng et al., 2020). These conflicting results might be the consequence of different methodologies used. While we employed purified monocytes to generate macrophages in six days, Zheng and collaborators kept PBMC with M-CSF for four days and then for another 10 days when the cells became adherent (Zheng et al., 2020). Another important methodological difference is that our study used ELISA to detect cytokines while Zheng and collaborators analyzed mRNA by RT-PCR. As the levels of mRNA induction found were rather low (fold change increase below 0.3), it is possible that protein detection by ELISA would have been below the detection limit as well. We are therefore proposing that hMDM generated from pure monocytes are not permissive to SARS-CoV-2 infection and do not mount inflammatory responses. This is in contrast to HCoV-229E and the TLR ligand controls. While future studies expanding to tissue macrophages are important, our results indicate that proinflammatory responses observed during COVID-19 may not be the result of macrophage infection but rather originate from other innate immune cells or a complex interaction between different immune cells which could include macrophages. Therefore, to understand these events more immune cells such as plasmacytoid dendritic cells that are at the frontline of the antiviral cytokine responses should be investigated.

In view of a potential link between ADE and inflammation during COVID-19 in the presence of antibodies, we tested this hypothesis using hMDM. With the selected sera from convalescent COVID-19 patients and the described conditions, even at very high serum dilutions and with sera that had low levels of neutralizing antibodies, we did not find evidence for antibody mediated enhancement of macrophage infection or pro-inflammatory cytokine responses. Interestingly, sera of COVID-19 convalescent patients showed cross-reactivity to SARS-CoV (Zettl et al., 2020), despite that the sera did not enhance infection of hMDM by this virus. This is in line with a previous report showing that the presence of cross-reacting antibodies against SARS-CoV-2, originating from previous endemic coronavirus infections, were not linked with more severe COVID-19 (Ng et al., 2020). Furthermore, in vaccination/challenge experiments carried out in macaques no signs of enhanced disease were detected (Gao et al., 2020; Yu et al., 2020). Finally, COVID-19 patients treated with plasma transfusion from convalescent patients did not show signs of disease aggravation (Casadevall and Pirofski, 2020; Joyner et al., 2020). Altogether, these data are in line with our findings, demonstrating that hMDM are not infected or activated by SARS-CoV-2 neither by direct contact nor mediated by antibodies from convalescent COVID-19 patients. Although the lack of hMDM infection by SARS-CoV and SARS-CoV-2 provides evidence of a lack of ADE, our study cannot exclude ADE effects on other FcR-expressing cells as well as a possible role of the complement system or T-cell mediated inflammation.

## Data Availability Statement

The original contributions presented in the study are included in the article/**Supplementary Material**. Further inquiries can be directed to the corresponding author.

## Ethics Statement

Ethical review and approval was not required for the study on human participants in accordance with the local legislation and institutional requirements. Written informed consent for participation was not required for this study in accordance with the national legislation and the institutional requirements.

## Author Contributions

OG-N, PV’K, and AS: conceptualization. OG-N, PV’K, FZ, GZ, VT and AS: methodology. OG-N, PV’K and FZ: investigation. OG-N, PV’K, FZ and AS: formal analysis. AS: supervision. OG-N and AS: writing–original draft. OG-N, PV’K, FZ, GZ, VT and AS: writing–review and editing. All authors contributed to the article and approved the submitted version.

## Conflict of Interest

The authors declare that the research was conducted in the absence of any commercial or financial relationships that could be construed as a potential conflict of interest.

## Supplementary Material

Supplementary Figure 1 Positive control experiment for ADE with hMDM. Serum from pig vaccinated with an experimental JEV vaccine (red) and naïve serum (black) were ten-fold diluted (from 1:10 to 1:10000) and incubated with JEV Laos at MOI of 1 TCID50/ml for 30, then immune complexes were incubated with hMDM for another 30 min, after wash and incubation for 24 h, infected cells were quantified by flow cytometry. Data from four independent experiments run in triplicates are shown. Each symbol represents the average value of infection in hMDM from a different donor. Statistically significant differences are indicated by asterisks (*p < 0.05, **p ≤ 0.002, and ***p ≤ 0.001).
