# Supplementary figures and images for: No Evidence for Human Monocyte-Derived Macrophage Infection and Antibody-Mediated Enhancement of SARS-CoV-2 Infection
Source: Front Cell Infect Microbiol. 2021 Apr 12;11:644574. doi: 10.3389/fcimb.2021.644574 (PMC8072125; doi:10.3389/fcimb.2021.644574)

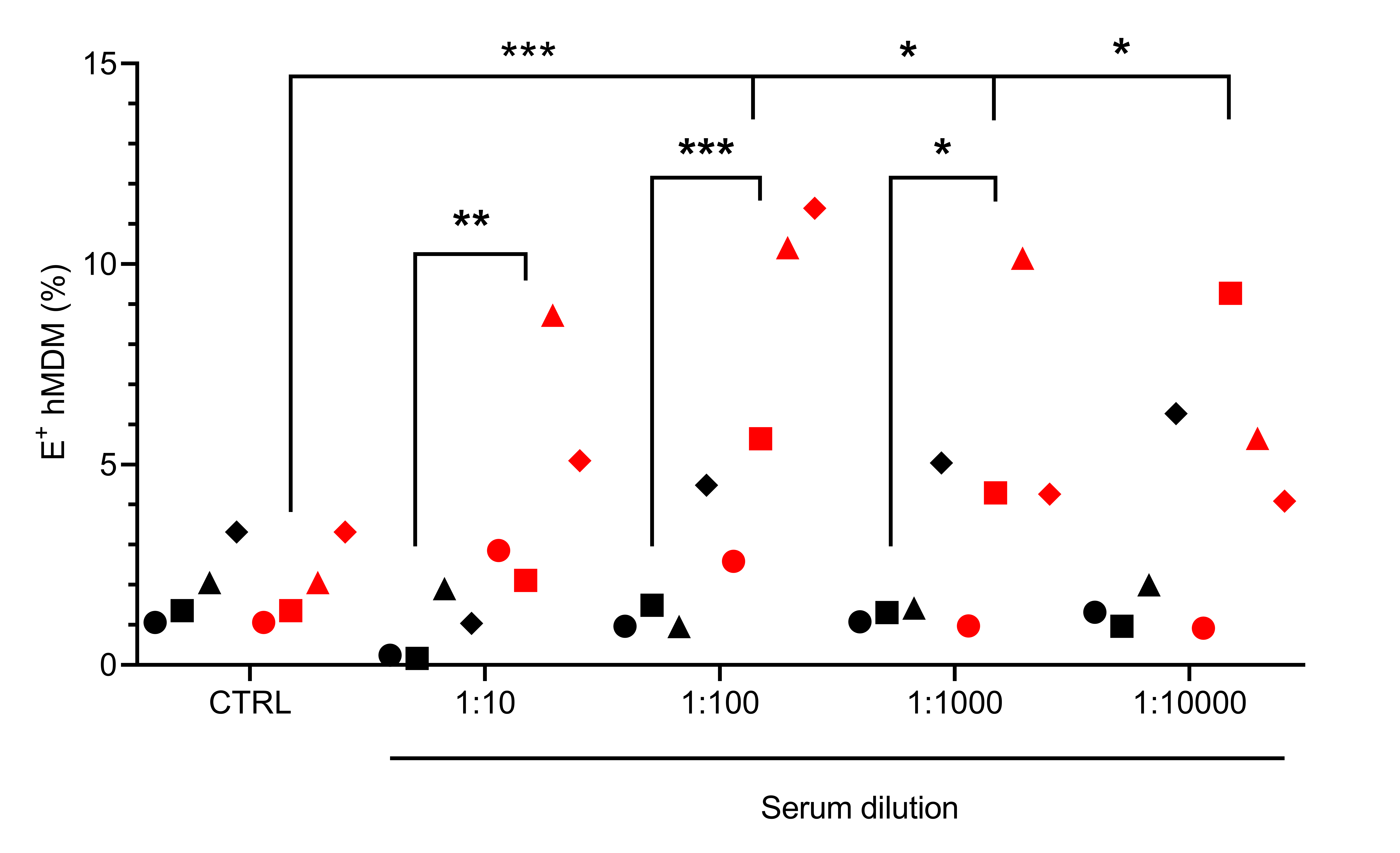

Supplement: Supplementary Figure 1 — Positive control experiment for ADE with hMDM. Serum from pig vaccinated with an experimental JEV vaccine (red) and naïve serum (black) were ten-fold diluted (from 1:10 to 1:10000) and incubated with JEV Laos at MOI of 1 TCID50/ml for 30, then immune complexes were incubated with hMDM for another 30 min, after wash and incubation for 24 h, infected cells were quantified by flow cytometry. Data from four independent experiments run in triplicates are shown. Each symbol represents the average value of infection in hMDM from a different donor. Statistically significant differences are indicated by asterisks (*p < 0.05, **p ≤ 0.002, and ***p ≤ 0.001). [file Image_1.tif]
